# Introduction to the Study on Regeneration in Lizards as an Amniote Model of Organ Regeneration

**DOI:** 10.3390/jdb9040051

**Published:** 2021-11-22

**Authors:** Lorenzo Alibardi

**Affiliations:** 1 Comparative Histolab Padova, 35100 Padova, Italy; lorenzo.alibardi@unibo.it; 2 Department of Biology, University of Bologna, Via Selmi 3, 40126 Bologna, Italy

## 1. The Lizard Model: Brief Historical Notes

Initial observations on the regeneration of the tail in lizards were recorded in brief notes by Aristotle over 2000 years ago, as reported in his book, *History of Animals* (cited from [1]). Only in a brief communication at the Paris Academy of Science in 1686, presented by M. Thevenot, could we find another mention of lizard tail regeneration. This was followed by a report from C. Perrault in 1688, indicating that the new tail derives from the growth of a pre-formed germ present in the tail [1].

**Figure 1:**
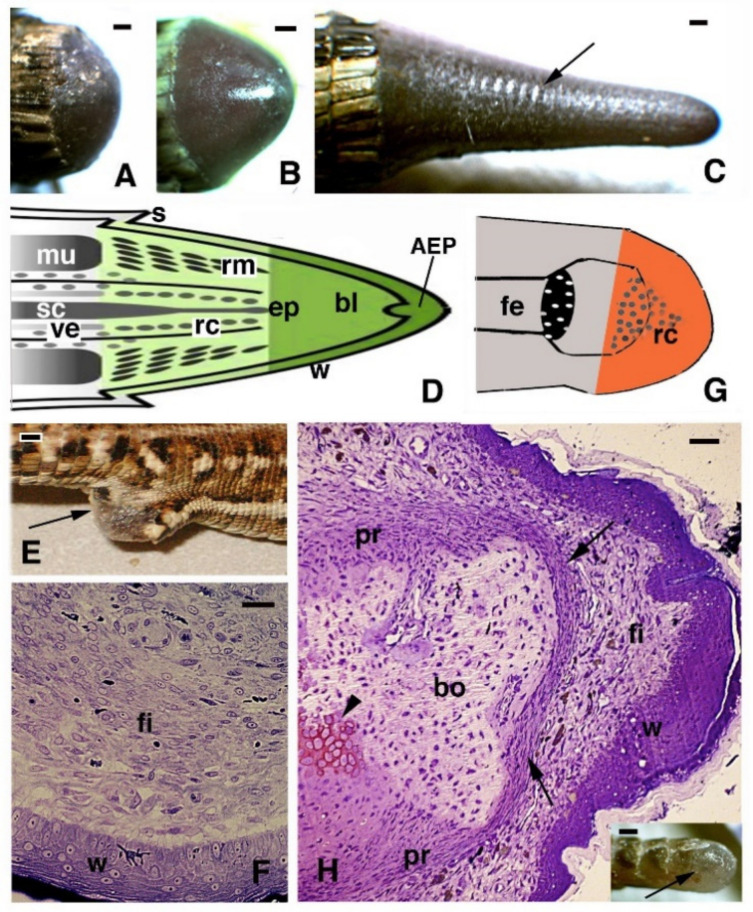
Images of a regenerating tail (**A**–**D**), limb (**E**–**G**), and finger (**H**) of the *Podarcis muralis* lizard. (**A**–**C**) Successive stages of regeneration from a blastema (**A**), a growing cone (**B**), and an elongating new tail with the beginning of scale formation (**C**), arrow. Bars represent 0.5 mm. (**D**) Schematic drawing that illustrates the main tissues present in the regenerating tail (green) as continuation of the stump (grey). (**E**) Scarring limb outgrowth (arrow) after about 25 days post-amputation. Bar, 1 mm. (**F**) Numerous fibroblasts are formed underneath the thick wound epidermis in limb blastema at 16 days post-amputation. Bar, 10 μm. (**G**) Schematic drawing of scarred limb outgrowth (orange). (**H**) Histological aspect of scarring finger at 20 days post-cut, containing a dense fibrocyte connective underneath a thick wound epidermis. Arrows indicate the formed dense periosteum covering the end of the phalange. The arrowhead indicates regenerated cartilage. Bar, 20 μm. The inset (bar 0.5 mm) shows the short scar (arrow) formed at the amputated digit tip. Legends: AEP, apical epidermal peg; bl, blastema; bo, bone (phalange); ep, ependymal tube; fe, femur with epiphyses; fi, fibroblasts/fibrocytes; mu, original muscles; pr, periosteum; rc, regenerating cartilage; rm, regenerating muscles; s, scale; sc, spinal cord; ve, vertebra.

The use of microscopes after cell theory from 1840 enabled initial microscopic observations on the process of regeneration after loss of the tail to be performed, mainly in European lizards. In 1885, Fraisse summarized the numerous studies carried out in this period, especially on the limited regeneration of nervous tissues in the new tail [2]. Due to the linguistics and difficulties in collecting papers or references from Asia, East Europe, and Russia, we can only trace the modern history of this research topic to the most available resources, recalling that studies from 1850 to 1950 were mainly performed by western Europeans, such as the Germans, Italians, French, and British. Scant information has been derived from Russian [3,4] and Chinese [5] studies, but this may have derived from languages and the difficulties retrieving other references from studies performed in these countries. These initial researchers provided detailed information on the progressive phases of tail regeneration over different species of lizards, mainly lacertids and geckos. Extensive references for these studies have been listed in Bellairs and Bryant (1985) and in Alibardi (2010) [6,7]; here, we only report on some of these studies which were carried out in specific aspects. It was soon discovered that the new tail, despite the massive regeneration of muscles and cartilage, is a large but simplified form of the tail in comparison to the original [2,8,9,10,11,12,13] (Figure 1A–D). The lack of a vertebral column and neuronal regeneration into a stratified SC is bypassed by innervation of the new tail from proximal regions of the spinal cord and ganglia, the cells of which become hypertrophic [14,15].

Aside from European investigators, numerous studies on the regenerating tail of lizards were carried out by Indian researchers between the late 1960s and 1980s (references in [6,7,16,17]). In addition to histological and histochemical studies, these investigators also conducted numerous biochemical analyses and enzymatic studies to obtain a broad picture of the metabolism of regenerating tails in geckos and scincid lizards. Several other histological and histochemical studies have been derived from African, Australian, and Caribbean geckos and scincids [18,19,20,21], and from the New Zealand tuatara [11,22,23,24,25].

Rare studies have instead been carried out on the amputated limbs and fingers of lizards, revealing that only scarring or limited heteromorphic regeneration takes place in these appendages [3,4,7,26,27,28,29,30,31,32]. Lizard regeneration research was carried out mainly by U.S. investigators, who took over the field from 1960 to 1995. Researchers in the United States were the first to use autoradiography and electron microscopy to analyze the regeneration of the nervous system and muscles of the tail [33,34,35,36,37,38,39,40], and also carried out in vitro studies on muscle differentiation [41,42]. These studies showed the kinetics of tissue formation in the regenerating tail.

I initiated my own studies on lizard regeneration around 1977, carrying out descriptive and experimental microscopic, histochemical, and biochemical studies on various lizards and geckos. Later, this research mainly concentrated on ultrastructural, autoradiographic, and immunohistochemical analyses of the process of tail and limb regeneration. These studies revealed, for the first time, the progressive stages of activation and transformation of cells from different tissues of the tail stump in ultrastructural detail, which gives rise to cells of the regenerative blastema that later differentiate into diverse tissues of the regenerated tail. Study of the regenerating spinal cord, represented by a thin ependymal tube, also revealed the differentiation of a few peculiar neurons, indicated as cerebrospinal fluid contacting neurons. These morphological and biochemical studies were synthesized in two small books [7,16]. I also tested the regenerative ability of other organs in lizards, in particular, the vertebrae and the articular cartilage of knees, in addition to attempts to stimulate regeneration of the limb using growth factors. Study on scale morphogenesis in the regenerating tail led, for the first time, to the identification of genes coding for the main proteins of the epidermis in the lizard species *Podarcis sicula* [43]. In particular, the first three beta-keratins ever sequenced, now indicated as corneous beta proteins (CβPs), have also enabled determination of the gene and protein structure in CβPs of all other reptiles: snakes, turtles, crocodilians, and the tuatara [44,45,46].

From 2005 onward, different groups from China [47,48,49], Canada [50,51,52,53,54], the United States [55,56,57,58,59,60,61,62], and India [63,64,65] have produced a series of studies on the biomolecular and immunohistochemical detection of numerous proteins during lizard regeneration. However, the era of molecular biology studies on the regeneration of the tail in lizards initiated with pioneering studies by the Kusumi group at Arizona State University in Phoenix, USA, who produced the first transcriptome of coding and non-coding genes of the regenerating tail in the lizard *Anolis carolinensis* [66,67]. This initial molecular study was rapidly followed by determination of the regenerating tail transcriptome in the Japanese gecko, *Geko japonicus* [68]. Further progress in the identification of key genes implicated in lizard regeneration was derived from the comparative analysis of the transcriptomes of the tail (regenerating) versus that of the limb (scarring) in the same lizard, the wall lizard *Podarcis muralis* [69,70]. The comparison between two different expression programs located in two different regions of the same animal, one destined to regenerate (the tail) and the other to scar (the limb), allowed the main genes orchestrating the regeneration of the tail to be determined, both coding and non-coding. A subsequent study on the expression of the main genes in regeneration compared to scarring tails confirmed that wnt2b, wnt6, c-myc, egfl6, and arhgap28 are among the main genes stimulating tail regeneration [71]. The study has continued until today, with the immunodetection of numerous coded proteins in both the tail and limbs (summarized in [32,72,73,74,75]). As a result of the above studies on lizards in comparison to fish and amphibian regeneration genes, I elaborated a new evolutionary explanation for the lack of organ regeneration in terrestrial vertebrates [32,74].

In the following chapter, a brief description of the process is provided to better introduce data shown in the following chapters of this Special Issue in *JDB*; however, further information is reported in other extensive reviews [7,50,51,76].

## 2. General Histological Aspects

**Figure 2:**
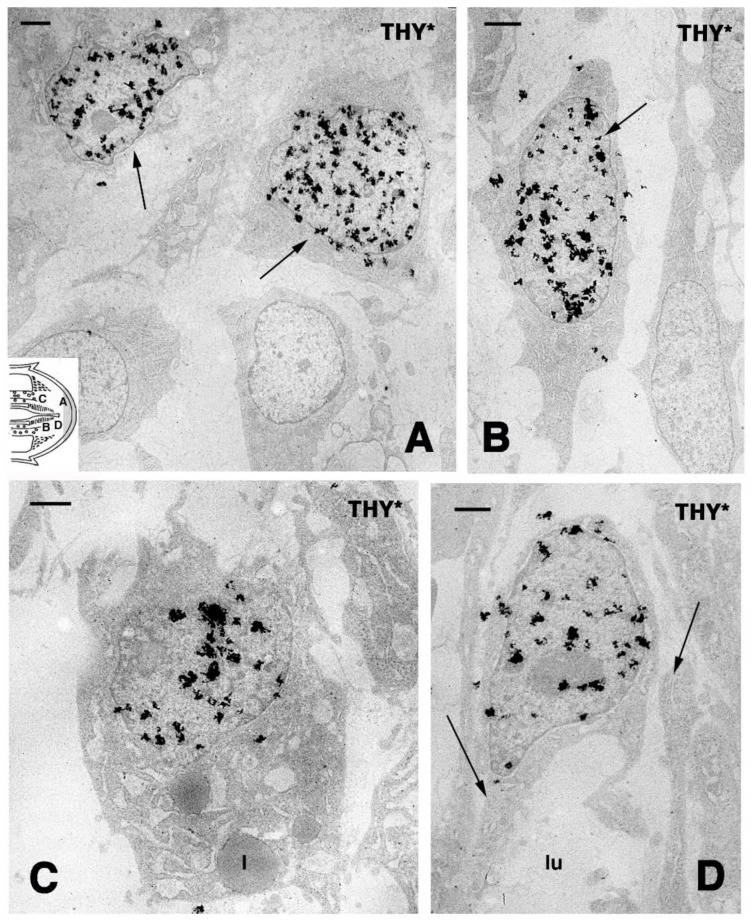
TEM autoradiography of blastema cells 4 h after injection of tritiated thymidine in *Anolis carolinensis*, showing nuclear labeling among different types of proliferating cells localized in the blastema (indicative positions shown by the letters in the inset drawing). (**A**) Mesenchymal cells with labeled nuclei (arrows) in the apical region near the epidermis. Bar, 1 μm. (**B**) Elongated fibroblast with labeled nucleus (arrow) detected in front of a vertebra fragment in the stump. Bar, 1 μm. (**C**) Labeled cells storing lipid droplets (l) and localized near the peri-vertebral fat layer of the tail stump. Bar, 1 μm. (**D**) Labeled endothelia cells (arrows point the vessel wall) of the forming capillary (lu, lumen) inside the blastema. Bar, 1 μm.

**Figure 3:**
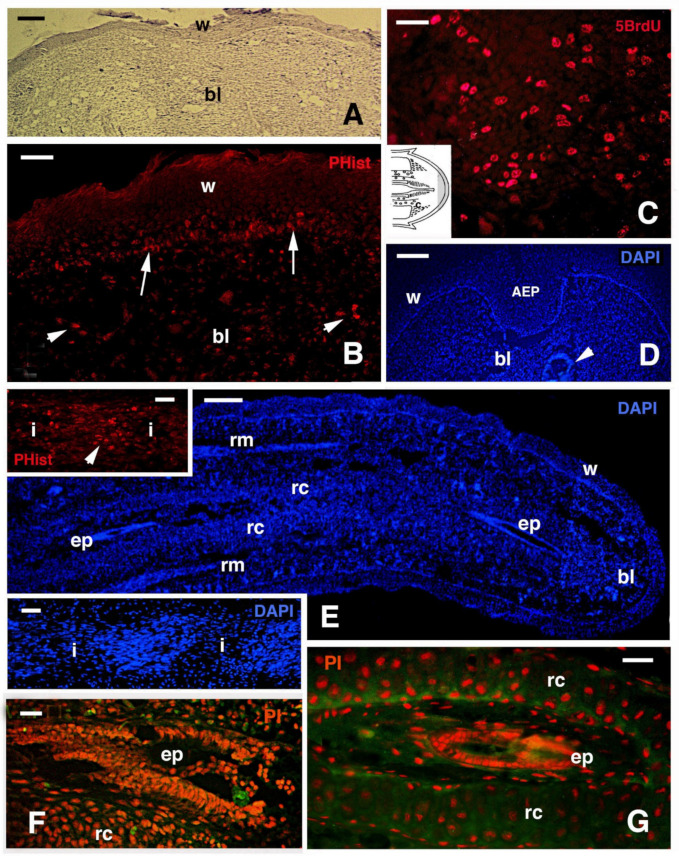
Histology (**A**) and fluorescence (**B**–**G**) of regenerating tails in *Podarcis muralis*. (**A**) Early blastema with covering wound epidermis at about 10 days post-amputation. Hemallume staining. Bar, 50 μm. (**B**) Area of the blastema showing distribution in the wound epidermis (arrows) and the mesenchyme (arrowheads) of labeled cells for the proliferative marker Phospho-histone (PHhis). Bar, 20 μm. (**C**) Detail on the numerous labeled nuclei for 5BrdU that are localized at the base of the blastema, near stump muscles (see (**C**) in the inset drawing). Bar, 10 μm. (**D**) Close-up of the tip of a regenerating cone with the apical epidermal peg stained with the fluorescent DAPI dye for nuclei. Bar, 50 μm. (**E**) Elongating tail showing the main tissues. Bar, 100 μm. The upper inset (Bar, 10 μm) shows labeled nuclei (arrowhead) for PHhistone-marker in proliferating cells (myoblasts). The lower inset (Bar, 10 μm), stained with DAPI, shows the formation of segmental myotomes. (**F**) Apical ependymal canal surrounded by early differentiating cartilaginous cells and stained with propidium iodide (nuclei). Bar, 10 μm. (**G**) Detail of the more proximal ependymal canal surrounded by differentiated chondrocytes forming a cartilaginous tube. Bar, 20 μm. Legends: AEP, apical epidermal cap; bl, blastema; ep, ependyma; i, forming inter-muscle connective tissue; rc, regenerating/-ed cartilage; rm, regenerating muscles; w, wound epidermis.

Despite the anatomical differences between the tail and limb, initially, an accumulation of mesenchymal-like cells occurs over the stumps of both the tail and limb, with blood cells of different types. Differently from the tail, the limbs and fingers undergo massive tissue destruction and inflammation and form a dense connective tissue 20–30 days after the amputation, while the epidermis rapidly becomes scaled and thick (Figure 1E–G). In the amputated limb, numerous cells accumulating on the stump surface are granulocytes and macrophages that maintain an intense and lasting inflammation for 2–3 weeks. In contrast, in the tail stump and early blastema, after 5–10 days, the hematogenous population is mainly replaced with proliferating mesenchymal cells (Figure 2 and Figure 3A–C). Although blastema cells under light microscopy appear as fibroblasts or mesenchymal cells, their ultrastructure and immunolabeling has sometimes revealed cytological details, suggesting their initial differentiation as muscle cells, connective fibroblasts, and cartilaginous or fat cells. Numerous discontinuous or fenestrated capillaries are also derived from intense angiogenesis (Figure 2).

**Figure 4:**
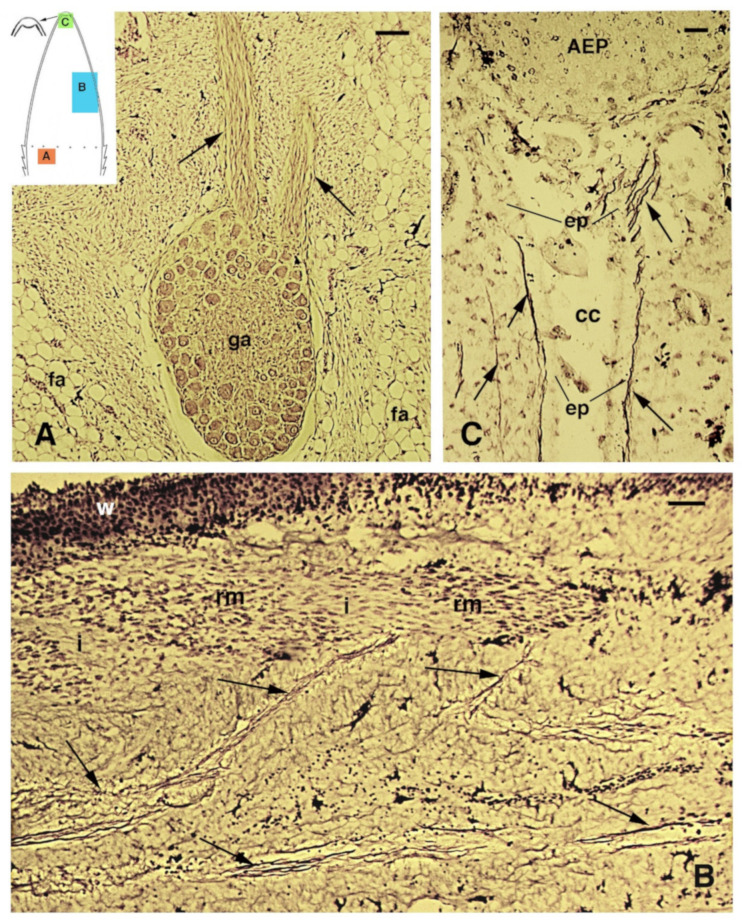
Bodian staining of spinal ganglia (**A**) and nerves (**B**,**C**) innervating the regenerating tail in *P. muralis*. (**A**) Ganglion with two main nerve bundles (arrows) directed into the elongating cone (15 days post-autotomy), and the position of which is indicated in the drawing (**A**). Bar, 50 μm. (**B**) Numerous nerves (arrows) innervate the regenerating tail (see the indicative position in (**B**) of the drawing), including the regenerating segmental muscles. Bar, 20 μm. (**C**) Detail of the few nerves reaching the apical ependymal ampulla that is present underneath the apical epidermal peg (indicative position in (**C**) of the drawing). Bar, 10 μm. Legends: AEP, apical epidermal peg; cc, central canal of the ependyma; ep, ependymal canal; fa, fat cells; ga, spinal ganglion; i, inter-muscular connective tissue; rm, regenerating muscles.

**Figure 5:**
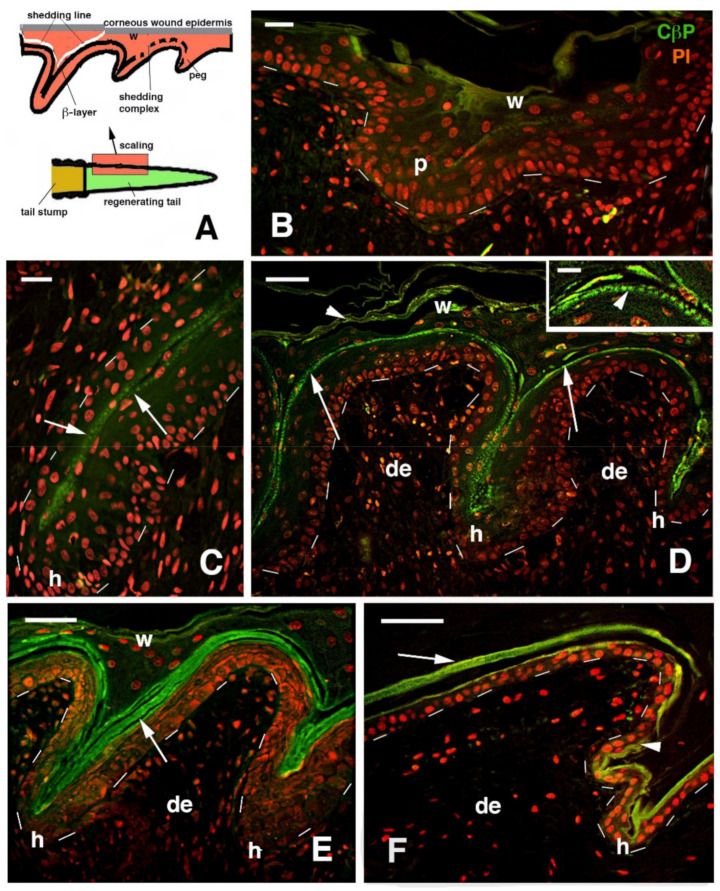
Drawing (**A**) and immunofluorescence for corneous beta proteins (CβP, green) and nuclear fluorescence from propidium bromide (red) in regenerating scales of *P. muralis* (**B**–**F**). (**A**) Drawing illustrating the proximal–distal formation of scales and the detachment of the external corneous wound epidermis along the shedding line, producing a molt. (**B**) Initial formation of epidermal pegs in the distal epidermis of a regenerating tail. Bar, 10 μm. (**C**) Detail showing the initial formation of the clear oberhautchen layer in the middle (arrows) of an elongated peg. Bar, 10 μm. (**D**) Formation of 2 scales separated by a hinge region that evidences a weakly immunolabeled outer corneous layer of the wound epidermis (arrowheads) and, more intensely, the forming oberhautchen layer (see detail in the inset, bar, 5 μm). Bar, 20 μm. (**E**) Intense labeling (arrows) of the thick beta layer in regenerating scales that are at a more advanced stage of morphogenesis than the previous figure. Bar, 20 μm. (**F**) Regenerated scale after shedding of the wound epidermis, see (**A**), with a compacted and immunolabeled beta-layer (arrow) that also extends to the oberhautchen (arrowhead) present in the inner and hinge regions of the new scale. Bar, 20 μm. Legends: de, dermis p, peg; h, forming hinge regions (inter-scale); w, corneous layer of the wound epidermis. Dashes underline the epidermis.

At the tip of the regenerating blastema of the tail, an apical epidermal peg (AEP) is formed and appears to be essential to maintain the growth of the forming tail. The underlying mesenchyme and extracellular matrix contain a low-density ground material rich in hyaluronate. In a few days, the blastema becomes a conical outgrowth that elongates into a new tail (Figure 1A–D and Figure 3E). While a mesenchymal blastema remains in contact with the apical epidermal peg only by the tip of the regenerating tail (Figure 3D), in the more proximal area, numerous tissues are progressively formed. Centrally, the spinal cord gives rise to an ependymal tube while lateral groups of segmental muscles are formed (Figure 3E,F). Intense cell multiplication occurs, especially in forming cartilage and muscles which become organized into myomeres (Figure 3E and insets). Around the apical ependyma, a condensation of fibroblast-like cells gives rise to a cartilaginous tissue that completely surrounds the ependymal tube, forming the new axial skeleton of the regenerated tail, which lacks vertebrae (Figure 3E,G). Only occasional holes are formed along the cartilaginous tube where blood vessels and few nerves can exit, although most innervation of the new tail derives from the three more proximal spinal cord neuromeres and ganglia present in the stump [15,62,77] (Figure 4A). Peripheral nerves from spinal neurons and ganglia grow into the regenerating tail, initially forming large bundles that separate and mainly innervate the new muscles (Figure 4B). However, thin nerves continue as far as the tip of the regenerating tail, together with those surrounding the apical ependyma (Figure 4C). Externally, the skin forms new scales from the proximal to distal regions (Figure 1C and Figure 5A). Scale regeneration occurs with a process apparently different from their development in the embryo, because regenerating scales derive from inward migration of the regenerating (wound) epidermis into forming the dermis and originating pegs (Figure 5A,B). The pegs elongate inside the dermis and their central keratinocytes initiate to differentiate new corneous layers that initially accumulate alpha-keratins (intermediate filament keratins), and eventually, CβPs (beta-keratins) in the new oberhautchen, which shows that the shedding line along with the external wound epidermis will be shed later, revealing the new scales formed underneath (Figure 5A,D). The subsequent layers of cells (beta) that differentiate beneath the oberhautchen accumulate a large amount of CβPs and the forming beta-layer appears intensely immunofluorescent (Figure 5C–F). The regenerated scales are shorter and more numerous than the original scales so that the regenerated tail can be recognized from the original tail, despite its size. These few notes indicate broad tissue regeneration with the recovery of numerous anatomical components of the tail into a very volumetric, albeit heteromorphic, organ.

## 3. Regeneration Evolved Only in Lizards, Providing Clues for Amniote Regeneration

It is still uncertain why only lizards, among amniotes, can regenerate a large organ, such as the tail, and why the other organs can only repair with much more limitation. A curious observation from a zoological and evolutionary standpoint is that eosuchians and primitive lizards with variably long tails are constantly depicted in paleontological and zoological books as the final/common victims of the other sauropsids of past and present times. Lepidosaurs in Permian–Triassic periods appeared to be victims of Mesozoic archosaurs, such as pterosaurs and dinosaurs, of ruling synapsids in Permian and therapsids in Jurassic, and later of the initial true mammals of the Jurassic–Cretaceous period. Presently, lizards are also the usual prey of snakes, birds, and small mammals, including domestic cats, to the point that sometimes one wonders how lizards are the most successful extant reptiles, with more than 3500 species, against 2700 for snakes, 200 for turtles, and 33 species of crocodilians [78]. For sure, one of the mechanisms essential for their evolutionary endurance has been tail autotomy, a process of self-amputation of the tail that is very effective to save a lizard’s life against predators [79,80,81].

As previously indicated [7,32,81], the loss of the tail, a non-vital organ but one with very useful ecological functions (running, climbing, fat storage, antipredator distractor, social display, etc.), was probably under selective pressure for tail regeneration. This is related to the development of autonomous planes where stem cells can be stored, as well as in the bony marrow where stem cells are activated after tail breakage. The observation that tail amputation in embryos determines an inability to regenerate [26,82] suggests that erasing the entire tail bud in early embryos will also erase stem cells and the regenerative potential of the definitive tail [7].

A regenerative blastema forms when a temporary immunosuppression is in place after injury or amputation [49,73,74,75]. Multiple factors contributed to the origin of an immune-privileged blastema: the production of antimicrobial peptides, synthesis of high levels of hyaluronate, initial production of immature anergic lymphocytes, Tregs lymphocytes, and M2-like macrophages, are all combined characteristics that maintain low inflammation and favor regeneration. The tail is not an immediately vital organ after loss in lizards; therefore, we can say that “natural selection worked on these different aspects”, lowering inflammation, and creating growth-factor-producing tissues (AEP and regenerating SC) and a gradient of genes that create a hyaluronate/highly hydrated and water/microbe-proof blastema capable of growth in the dry conditions of terrestrial life. Lizard-regenerating blastema provides clues on mechanisms for the control of cell proliferation and growth in amniote organs without degenerating into teratomes or cancer. An interesting aspect is that whereas the process of scarring in lizard limbs and fingers resembles that of mammals, the tail instead forms a true, albeit simplified, embryonic organ. Therefore, the lizard model of regeneration is also useful for studies on the process of scarring versus regeneration in mammalian organs. The molecular information derived from the knowledge of this model for regenerative medicine might serve useful in the discovery of antimicrobe peptide drugs [83,84], molecules for the control of inflammation and scarring, molecules for the control of cell proliferation in cancer, the development of technological and medical procedures for creating regenerative outgrowths, and bio-prostheses for limb replacements.

## References

[1] Dinsmore C.H. (1996). Urodele limb and tail regeneration in early biological thought: An essay on scientific controversy and social change. Int. J. Dev. Biol..

[2] Fraisse P. (1885). Die Regeneration von Geweben und Organen bei den Wirbeltieren Besonders bei Amphibien und Reptilien.

[3] Kudokotsev V.P. (1960). Regeneration process of extremity in lizard, stimulated by the method of supplementary innervation. Dokl. Akad. Sci. SSSR.

[4] Kudokotsev V.P. (1960). Regeneration of the limbs in the dersert snake-eyed skink (*Ablepharus deserti* Strauch). Dokl. Akad. Sci. SSSR.

[5] Boring A.M., Lan-Fen C., Wei-His C. (1948–1949). Autotomy and regeneration in the tail of lizards. Peking Natur. Hist. Bull..

[6] Bellairs A.D.A., Bryant S.V., Gans C., Billet F., Maderson P.F.A. (1985). Autotomy and regeneration in reptiles. Biology of the Reptilia.

[7] Alibardi L. (2010). Morphological and cellular aspects of tail and limb regeneration in lizard: A model system with implications for tissue regeneration in mammals. Adv. Anat. Embryol. Cell Biol..

[8] Calori L. (1858). Sullo scheletro della *Lacerta viridis* Linn., sulla riproduzione della coda nelle lucertole, e sulle ossa cutanee del teschio dei sauri. Mem. Atti. Acc. Sci. Bologna..

[9] Giuliani M. (1878). Sulla struttura del midollo spinale. Sulla riproduzione della coda della *Lacerta viridis*. Ric. Lab. Anat. Norm..

[10] Misuri A. (1910). Ricerche sulla struttura della coda normale e rigenerata nella *Lacerta muralis*. Memor. Boll. Soc. Zool. Ital..

[11] Woodland W.N.F. (1920). Some observations on caudal autotomy and regeneration in the gecko (*Hemidactylus flaviviridis*, Ruppel), with notes on the tails of *Sphenodon* and *Pygopus*. Quart. J. Microsc. Sci..

[12] White C.P. (1925). Regeneration of the lizard’s tail. J. Path. Bact..

[13] Quattrini D. (1954). Piano di autotomia e rigenerazione della coda nei Sauri. Arch. Ital. Anat. Embr..

[14] Terni T. (1915). Studio anatomico di una coda doppia. Arch. Ital. Anat. Embryol..

[15] Terni T. (1920). Sulla correlazione fra amplezza del territorio di innervazioni e grandezza della cellule gangliare. 2. Richerche sui gangli spinali che innervano la coda rigenerata, dei Sauri (*Gongylus ocellatus*). Arch. Ital. Anat. Embriol..

[16] Alibardi L. (2014). Histochemical, Biochemical and Cell Biological aspects of tail regeneration in lizard, an amniote model for studies on tissue regeneration. Prog. Histochem. Cytoch..

[17] Ramachandran A.V. (2006). Biochemistry and metabolism of lizard tail regeneration. J. Anim. Morphol. Physiol..

[18] Hughes A., New D. (1959). Tail regeneration in the geckonid lizard, *Sphaerodactylus*. J. Embryol. Exp. Morphol..

[19] Werner Y.L. (1967). Regeneration of the caudal axial skeleton in a gekkonid lizard (*Hemidactylus*) with particular reference to the “latent” period. Acta Zool..

[20] Magon D.K. (1977). Glucose metabolism in the regenerating tail of the scincid lizard, *Mabuya striata*: Glycogen, phosphorylase and aldolase activity. J. Nat. Hist..

[21] Purvis M.D. (1979). Early stages of tail regeneration in *Lampropholis guichenoti*. Aust. Zool..

[22] Byerly T.C. (1925). Note on the partial regeneration of the caudal region of *Sphenodon punctatus*. Anat. Rec..

[23] Ali S.M. (1941). Studies on the comparative anatomy of the tail in sauria and rhynchocephalia *Sphenodon punctatus* Gray. Proc. Ind. Acad. Sci..

[24] Alibardi L., Meyer-Rochow V.B. (2019). Microscopical observations on the regenerating tail in the tuatara *Sphenodon punctatus* indicate a tendency to scarring, but also influence from somatic growth. J. Morph..

[25] Alibardi L., Meyer-Rochow V.B. (2021). Regeneration in Reptiles Generally and the New Zealand Tuatara in Particular as a Model to Analyze Organ Regrowth in Amniotes. J. Dev. Biol..

[26] Marcucci E. (1915). Gli arti e la coda della *Lacerta muralis* rigenerano nello stadio embrionale?. Boll. Soc. Natural..

[27] Marcucci E. (1930). Il potere rigenerativo degli arti nei Rettili. Ricerche sperimentali sopra alcune specie di Sauri. Arch. Zool. Ital..

[28] Marcucci E. (1930). La rigenerazione nei rettili. Archivio Zool. Ital..

[29] Guyénot E., Matthey R. (1928). Les processus régénératifs dans la patte posterieure du lezard. Wilhelm Roux’Archiv Entwick. Organ..

[30] Barber L.W. (1944). Correlations between wound healing and regeneration in fore-limbs and tails of lizards. Anat. Rec..

[31] Bellairs A.D.A., Bryant S.V. (1968). Effects of amputation on limbs and digits of lacertid lizards. Anat. Rec..

[32] Alibardi L. (2021). Tail regeneration in lepidosauria as an exception to the generalized lack of organ regeneration in amniotes. J. Exp. Zool. Part B Mol. Dev. Evol..

[33] Simpson S.B., Kiortsis V., Trampusch H.A.L. (1965). Regeneration of the lizard tail. Regeneration in Animals and Related Problems.

[34] Simpson S.B. (1968). Morphology of the regenerated spinal cord in the lizard *Anolis carolinensis*. J. Comp. Neur..

[35] Zika J., Singer M. (1965). The relation between nerve fiber number and limb regenerative capacity in the lizard, Anolis. Anat. Rec..

[36] Cox P.G. (1969). Some aspects of tail regeneration in the lizard, *Anolis carolinensis*. I. A description based on histology and autoradiography. J. Exp. Zool..

[37] Egar M., Simpson S.B., Singer M. (1970). The growth and differentiation of the regenerating spinal cord after autotomy of the lizard *Anolis carolinensis*. J. Morph..

[38] Simpson S.B., Bayne E.K., Mauro A. (1979). In vivo and in vitro studies of regenerating muscle in the lizard Anolis. Muscle Regeneration.

[39] Kahn E.B., Simpson S.B. (1974). Satellite cells in mature, uninjured skeletal muscle of the lizard tail. Dev. Biol..

[40] Simpson S.B., Duffy M.T. (1994). The lizard spinal cord: A model system for the study of spinal cord injury and repair. Progr. Brain Res..

[41] Chlebowsky J.S., Przybylski R.J., Cox P.G. (1973). Ultrastructural studies of lizard (*Anolis carolinensis*) myogenesis in vitro. Dev. Biol..

[42] Marusich M.F., Simpson S.B. (1983). Changes in cell surface antigens during in vitro lizard myogenesis. Dev. Biol..

[43] Dalla Valle L., Toffolo V., Belvedere P., Alibardi L. (2005). Isolation of a mRNA encoding a glycine-proline-rich beta-keratin expressed in the regenerating epidermis of lizard. Dev. Dyn..

[44] Alibardi L., Dalla Valle L., Nardi A., Toni M. (2009). Evolution of hard proteins in the sauropsid integument in relation to the cornification of skin derivatives in amniotes. J. Anat..

[45] Holthaus K.B., Eckhart L., Dalla Valle L., Alibardi L. (2019). Evolution and diversification of corneous beta-proteins, the characteristic epidermal proteins of reptiles and birds. J. Exp. Zool..

[46] Holthaus K.B., Alibardi L., Tschachler E., Eckhart L. (2020). Identification of epidermal differentiation genes of the tuatara provides insights into the early evolution of lepidosaurian skin. Sci. Rep..

[47] Wang Y., Wang R., Jang S., Zhou W., Liu Y., Gu Q., Gu Y., Dong Y., Liu M., Ding F. (2011). Gecko CD59 is implicated in proximodistal identity during tail regeneration. PLoS ONE.

[48] Zhou Y., Xu Q., Li D., Zhao L., Wang Y., Liu M., Gu X., Liu Y. (2013). Early neurogenesis during caudal spinal cord regeneration in adult *Gekko japonicus*. J. Mol. Histol..

[49] He B., Song H., Wang Y. (2021). Self-control of inflammation during tail regeneration of lizards. J. Dev. Biol..

[50] McLean C.E., Vickaryous M.K. (2011). A novel amniote model of epimorphic regeneration: The leopard gecko, *Eublepharis macularius*. BMC Dev. Biol..

[51] Gilbert E.A.B., Payne S.L., Vickaryous M.K. (2013). The anatomy and histology of caudal autotomy and regeneration in lizards. Physiolog. Bioch. Zool..

[52] Gilbert E.A.B., Delorme S.L., Vickaryous M.K. (2015). The regeneration blastema of lizards: An amniote model for the study on appendage replacement. Regeneration.

[53] Gilbert R.W.D., Vickaryous M.K., Victoria-Petit A.M. (2016). Signalling by transforming growth factor beta isoforms in wound healing and tissue regeneration. J. Dev. Biol..

[54] Gilbert E.A.B., Vickaryous M.K. (2018). Neural stem/progenitor cells are activated during tail regeneration in the leopard gecko (*Eleublepharis macularius*). J. Comp. Neurol..

[55] Fisher R.E., Geiger L.A., Stroik L.K., Hutchins E.D., George R.M., DeNardo D.F., Kusumi K., Rawls J.A., Wilson-Rawls J. (2012). A histological comparison of the original and regenerated tail in the green anole, *Anolis carolinensis*. Anat. Rec..

[56] Lozito T.P., Tuan R.S. (2015). Lizard tail regeneration: Regulation of two distinct cartilage regions by Indian hedgehog. Dev. Biol..

[57] Lozito T.P., Tuan S.R. (2016). Lizard tail regeneration as an instructive model of enhanced healing capabilities in an adult amniote. Connect. Tiss. Res..

[58] Lozito T.P., Tuan R.S. (2016). Lizard tail skeletal regeneration combines aspects of fracture healing and blastema-based regeneration. Development.

[59] Hutchins E.D., Wilson-Rawls J., Kusumi K., Wilson-Rawls J., Kusumi K. (2016). Regeneration: Lessons from lizards. Innovations in Molecular Mechanisms and Tissue Engineering, Stem Cell Biology and Regenerative Medicine.

[60] Londono R., Wenzhong W., Wang B., Tuan R.S., Lozito T.P. (2017). Cartilage and muscle cell fate and origin during lizard tail regeneration. Front. Bioeng. Biotech..

[61] Palade J., Djordjevic D., Hutchins E.D., George R.M., Cornelius J.A., Rawls A., Ho J.W.K., Kusumi K., Wilson-Rawls J. (2018). Identification of satellite cells from anole lizard skeletal muscles and demonstration of expanded musculoskeletal potential. Dev. Biol..

[62] Tokuyama M.A., Xu C., Fisher R.E., Wilson-Rawls J., Kusumi K., Newbern J.M. (2018). Developmental and adult-specific processes contribute to de novo neuromuscular regeneration in the lizard tail. Dev. Biol..

[63] Sharma P., Suresh B. (2008). Influence of Cox-2-induced PGE2 on the initiation and progression of tail regeneration in Northern house gecko, *Hemidactylus flaviviridis*. Folia Biol..

[64] Nambiar V.V., Bhatt I.Y., Deshmukh P.A., Jape D.D.J., Jivani P.N., Kavale H.R., Prakashkar S.S., Ramachandran A.V. (2008). Assessment of extracellular matrix remodeling during tail regeneration in the lizard Hemidactylus flaviviridis. J. Endocr. Reprod..

[65] Murawala H., Ranadive I., Patel S., Desai I., Balakrishnan S. (2018). Protein expression pattern and analysis of differentially expressed peptides during various stages of tail regeneration in *Hemidactylus flaviviridis*. Mech. Dev..

[66] Hutchins E.D., Markov G.J., Eckalbar W.L., Gorge R.M., King J.M., Tokuyama M.A., Geiger L.A., Emmert N., Ammar M.J., Allen A.P. (2014). Transcriptomic analysis of tail regeneration in the lizard *Anolis carolinensis* reveals activation of conserved vertebrate developmental and repair mechanisms. PLoS ONE.

[67] Hutchins E.D., Eckalbar W.L., Walter J.M., Mangone M., Kosumi K. (2016). Differential expression of conserved and novel microRNAs during tail regeneration in the lizard *Anolis carolinensis*. BMC Genom..

[68] Liu Y., Zhou Q., Wang Y., Luo L., Yang J., Yang L., Liu M., Li Y., Qian T., Zheng Y. (2015). Gekko japonicus genome reveal evolution of adhesive toe pads and tail regeneration. Nat. Commun..

[69] Vitulo N., Dalla Valle L., Skobo T., Valle G., Alibardi L. (2017). Transcriptome analysis of the regenerating tail versus the scarring limb in lizard reveals pathways leading to successful versus unsuccessful organ regeneration in amniotes. Dev. Dyn..

[70] Vitulo N., Dalla Valle L., Skobo T., Valle G., Alibardi L. (2017). Down-regulation of lizard immuno-genes in the regenerating tail and myo-genes in the scarring limb suggests that tail regeneration occurs in an immuno-privileged organ. Protoplasma.

[71] Degan M., Dalla Valle L., Alibardi L. (2021). Gene expression in regenerating and scarring tails of lizard evidences three main key genes (wnt2b, egfl6 and arhgap28) activated during the regulated process of tail regeneration. Protoplasma.

[72] Alibardi L. (2017). Biological and molecular differences between tail regeneration and limb scarring in lizard, an inspiring model addressing limb regeneration in amniotes. J. Exp. Zool. B.

[73] Alibardi L. (2017). Hyaluronic acid in the tail and limb of amphibians and lizards recreates permissive embryonic conditions for regeneration due to its hygroscopic and immuno-suppressive properties. J. Exp. Zool. B.

[74] Alibardi L. (2020). Appendage regeneration in anamniotes utilizes genes active during larval-metamorphic stages that have been lost or altered in amniotes: The case for studying lizard tail regeneration. J. Morphol..

[75] Alibardi L. (2021). Regeneration of the tail in lizards appears regulated by a balanced expression of oncogenes and tumor suppressors likely controlling epidermal mesenchymal transition and apical tail growth. Ann. Anat..

[76] Jacyniak K., McDonald R.P., Vickaryous M.K. (2017). Tail regeneration and other phenomena of wound healing and tissue restoration in lizards. J. Exp. Biol..

[77] Alibardi L. (2021). Spinal ganglia and peripheral nerves innervating the regenerating tail and muscles of lizards. J. Morphol..

[78] Pough H.F., Janis C.M., Heiser J.B. (2009). Vertebrate Life.

[79] Arnold N. (1990). The throwaway tail. New Sci..

[80] Ananieva N.B., Gordeev D.A., Korost D.V. (2021). The review of the autotomy of agamid lizards with considerations about the types of autotomy and regeneration. J. Dev. Biol..

[81] Bateman J.J., Boisvert C.A., Bateman P.W. (2021). At what cost? Trade-off and influences on energetic investment to tail regeneration in lizards following autotomy. J. Dev. Biol..

[82] Bryant S.V., Bellairs A.D.A. (1967). Amnio-allantoic constriction bands in lizard embryos and their effects on tail regeneration. J. Zool..

[83] Alibardi L. (2015). Immunolocalization indicates that both original and regenerated lizard tail tissues contain populations of long retaining cells, putative stem/progenitor cells. Micr. Res. Techn..

[84] Holthaus K.B., Spisni E., Alibardi L. (2016). Microbicide activity of two reptilian antimicrobial peptides on Gram positive and Gram negative bacteria. J. Immunobiol..

